# Trends in the Use of Driving-Impairing Medicines According to the DRUID Category: A Population-Based Registry Study with Reference to Driving in a Region of Spain between 2015 and 2019

**DOI:** 10.3390/ph16040508

**Published:** 2023-03-29

**Authors:** Eduardo Gutiérrez-Abejón, Paloma Criado-Espegel, M. Aránzazu Pedrosa-Naudín, Diego Fernández-Lázaro, Francisco Herrera-Gómez, F. Javier Álvarez

**Affiliations:** 1 Pharmacological Big Data Laboratory, Faculty of Medicine, University of Valladolid, 47005 Valladolid, Spain; 2 Pharmacy Directorate, Castilla y León Health Council, 47007 Valladolid, Spain; 3 Department of Cellular Biology, Histology and Pharmacology, Faculty of Health Sciences, Campus of Soria, University of Valladolid, 42003 Soria, Spain; 4 Neurobiology Research Group, Faculty of Medicine, University of Valladolid, 47005 Valladolid, Spain; 5 Transplantation Center, Faculty of Medicine, Lausanne University Hospital, University of Lausanne, CH-1011 Lausanne, Switzerland; 6 Department of Kidney Resuscitation and Acute Purification Therapies, Complejo Asistencial de Zamora, 49022 Zamora, Spain; 7 CEIm, Hospital Clínico Universitario de Valladolid, 47003 Valladolid, Spain

**Keywords:** driving-impairing medicines, automobile driving, drug utilization, traffic accidents, driving under influence, DRUID classification

## Abstract

The European DRUID (Drive Under the Influence of drugs, alcohol, and medicines) program classifies medications into three categories according to their effect on one’s fitness to drive. The trend in the use of driving-impairing medicines (DIMs) in a region of Spain between 2015 and 2019 was analyzed through a population-based registry study. Pharmacy dispensing records for DIMs are provided. The use of DIMs on drivers was weighted according to the national driver’s license census. The analysis was performed considering the population distribution by age and sex, treatment length, and the three DRUID categories. DIMs were used by 36.46% of the population and 27.91% of drivers, mainly chronically, with considerable daily use (8.04% and 5.34%, respectively). Use was more common in females than in males (42.28% vs. 30.44%) and increased with age. Among drivers, consumption decreases after 60 years of age for females and after 75 years of age for males. There was a 34% increase in the use of DIMs between 2015 and 2019, with a focus on daily use (>60%). The general population took 2.27 ± 1.76 DIMs, fundamentally category II (moderate influence on fitness to drive) (20.3%) and category III (severe influence on fitness to drive) (19.08%). The use of DIMs by the general population and drivers is significant and has increased in recent years. The integration of the DRUID classification into electronic prescription tools would assist physicians and pharmacists in providing adequate information to the patient about the effects of prescribed medications on their fitness to drive.

## 1. Introduction

According to the World Health Organization (WHO), injuries from traffic crashes will be the fifth leading cause of death by 2030, representing a serious public health problem worldwide [1].

Two of the most important factors to consider when assessing the risk of a traffic collision are age and sex. Older drivers have been shown to be up to 20 times more likely to be involved in a traffic crash than younger people [2], constituting the second leading cause of unintentional injury death for people over the age of 55 [3]. This is primarily because the skills needed to drive, such as attention, executive function, and visuospatial ability, decline with aging [4]. On the other hand, in terms of sex, males have higher risk values than females, especially at younger ages [5]. This may be due to several factors: females drive half as much as males [6] and show greater self-regulation behind the wheel, avoiding risky behaviors [7].

A significant proportion of these injuries can be attributed to driving after consuming psychoactive substances such as alcohol, illicit drugs, or certain medicines. Awareness of the role of these substances in driving is increasing, as is the implementation of appropriate interventions [8,9,10]. It is well known that alcohol and illicit drugs such as opioids, cocaine, and cannabis can impair a driver’s psychomotor performance [11,12,13,14].

Driving a motor vehicle is a complex task that requires different cognitive and psychomotor capacities. The ability to drive a vehicle depends on the vision, brain, and musculoskeletal system performing the tasks involved in coordinated driving [15]. Some of the most commonly prescribed medicines can impair visual, cognitive, and/or motor skills necessary for safe driving [16]. Several groups of medicines can impair psychomotor performance, while the relationship between medication use and the risk of being involved in a traffic crash has been analyzed in several studies [9,10,17]. This association is evident for benzodiazepines [18,19,20], as well as for other commonly prescribed medicines such as antipsychotics, antidepressants, anxiolytics, opioid analgesics, etc. [12,15,21,22,23,24]. According to the European DRUID (Driving Under Influence of Alcohol and Drugs) project, the third and second most detected substance in traffic crashes and fatalities, respectively, was a benzodiazepine [25].

Due to the association between medicines and potential driving impairment, the European Union (EU) requires pharmaceutical companies to provide data on the effects of each drug on driving ability before marketing [26]. This information warns of possible effects that drivers may experience (drowsiness, dizziness, blurred vision, etc.) and that may significantly impair their fitness to drive [27].

Thus, some countries have chosen to print a driving pictogram on the outer packaging of certain medicines to warn of the potential risks while driving [28]. Currently, many European countries such as Spain, France, Austria, Denmark, Finland, among others, includes pictograms on certain medicines, which it is legally binding [29,30,31].

However, the population does not receive adequate information about the effects of medicines on driving ability. In Spain, for example, a study revealed that only 15.9% of the population was aware of the existence of the “medicines and driving” pictogram. The study also showed that most patients did not receive adequate information from healthcare professionals (physicians and pharmacists) about the effects of medicines on driving [28].

To address the problem of misinformation and to assist healthcare professionals in the medication selection process, several approaches to categorize medicines according to their effect on driving have been conducted [25,32]. One of the most comprehensive classifications is provided by the European DRUID project. According to this classification, the medicines are distinguished into three categories based on their effects on one’s fitness to drive: I (minor influence), II (moderate influence) and III (severe influence) [27]. The definition of the DRUID categories was based on pharmacodynamic and kinetic data, pharmacovigilance data, experimental and epidemiological data, and additional data [27]. Considering these topics, the definitions of each DRUID category are shown in Supplementary Table S1 [27].

However, the DRUID classification does not include natural products that may affect psychomotor performance [33,34,35,36], which may be a handicap. In this sense, beneficial nutrients and antioxidants, including coenzyme Q10 (CoQ10) and alpha-tocopherol (α-Toc), and genistein proved to exhibit a neuroprotective effect attributed to its antioxidant, and/or anti-apoptotic properties [37,38] as well as α-Toc and/or CoQ10, where the underlying molecular mechanism of the treating compounds is related to the vascular endothelial growth factor (VEGF) and enhancing the brain-derived neurotrophic factor (BDNF)/tyrosine kinase B (TrkB)/cAMP-response element-binding protein (CREB) signaling pathway [39]. Additionally, the effects of the administration of the anti-amnestic dose of St. John’s wort extract and hyperforin on the ability to manage one’s cognitive disturbance in psychotic and Huntington’s disease patients were studied, among which the manifestation of prepulse inhibition (PPI) deficit was detected [40], wherein hyperforin was found to be one of the active ingredients responsible for St. John’s wort-induced PPI disruption with no relation to apoptotic processes [41].

Based on previous research conducted by our group, approximately one in four drivers in Spain uses driving-impairing medicines (DIMs), with chronic use being the most common [42]. Up to now, trends in the use of DIMs have been analyzed: benzodiazepines [43], antipsychotics [44], opioids [45], antidepressants [46], antidiabetics [47], and antihistamines [48]. Now, we aim to analyze the use trend of DIMs according to the DRUID classification.

This study presents data on the consumption of DIMs by each DRUID category (I, II, and III) between 2015 and 2019 in Castile and Leon, a region of Spain with 2,323,770 inhabitants. The length of treatment and concomitant use among the different DRUID categories were specially addressed. Similarly to previous manuscripts, an estimate of the use of these medications in drivers was made. Lastly, all data are reported by sex and age group.

## 2. Results

Approximately 57.5 million packages of DIMs were dispensed in Castile and Leon between 2015 and 2019. For every 10 packages dispensed, 8 were medicines in Group N (central nervous system) of the Anatomic Therapeutic Chemical (ATC) classification, and 1 was a medicine in Group A (alimentary tract and metabolism). For each DRUID category, the most commonly used medicines were: lorazepam (DRUID III, 4.464.023 packages), codeine, and paracetamol (DRUID II, 1.211.750 packages) and metamizole (DRUID I, 4.753.078 packages) (Supplementary Table S2).

**Figure 1:**
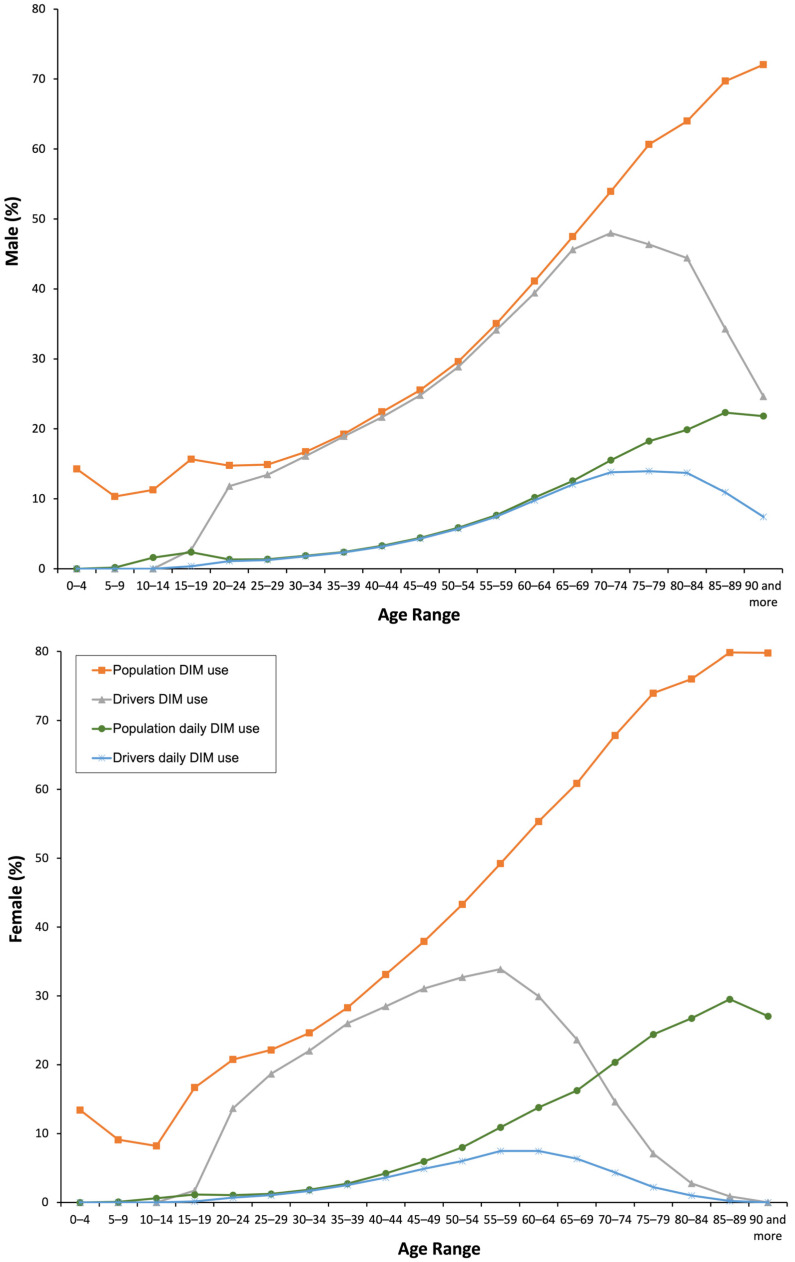
Frequency of the use of DIMs by the general population and the driver population.

**Figure 2:**
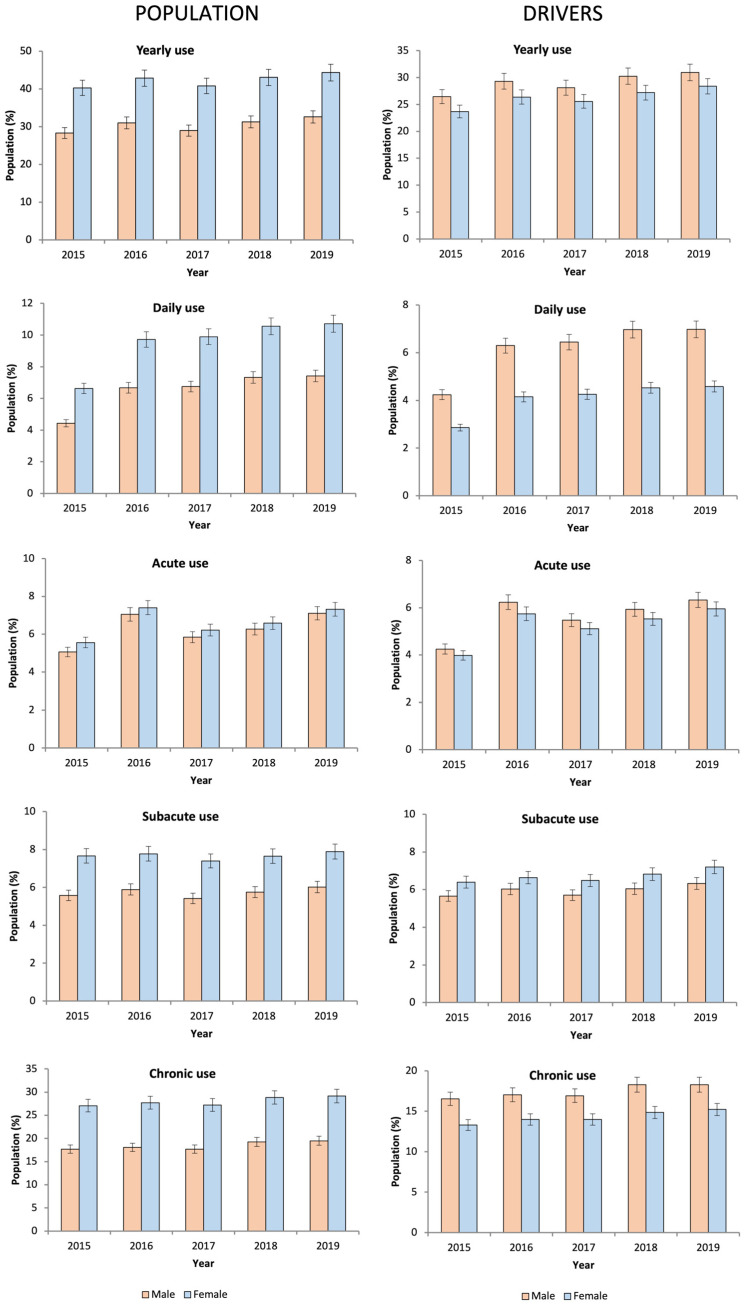
Evolution of the use of DIMs in Castile and Leon by the frequency of use (2015–2019).

Overall, 36.4% of the general population took at least one DIM, mainly anxiolytics (14.27%), opioids (11.75%), antidepressants (8.75%), and other analgesics/antipyretics (8.25%). Chronic use (23.3%) predominated over subacute (6.72%) and acute (6.44%) use. Females were more likely to use DIM than males (42.28% vs. 30.44%, *p* = 0.001), and the use increased with age (Table 1, Figure 1 and Figure 2).

**Figure 3:**
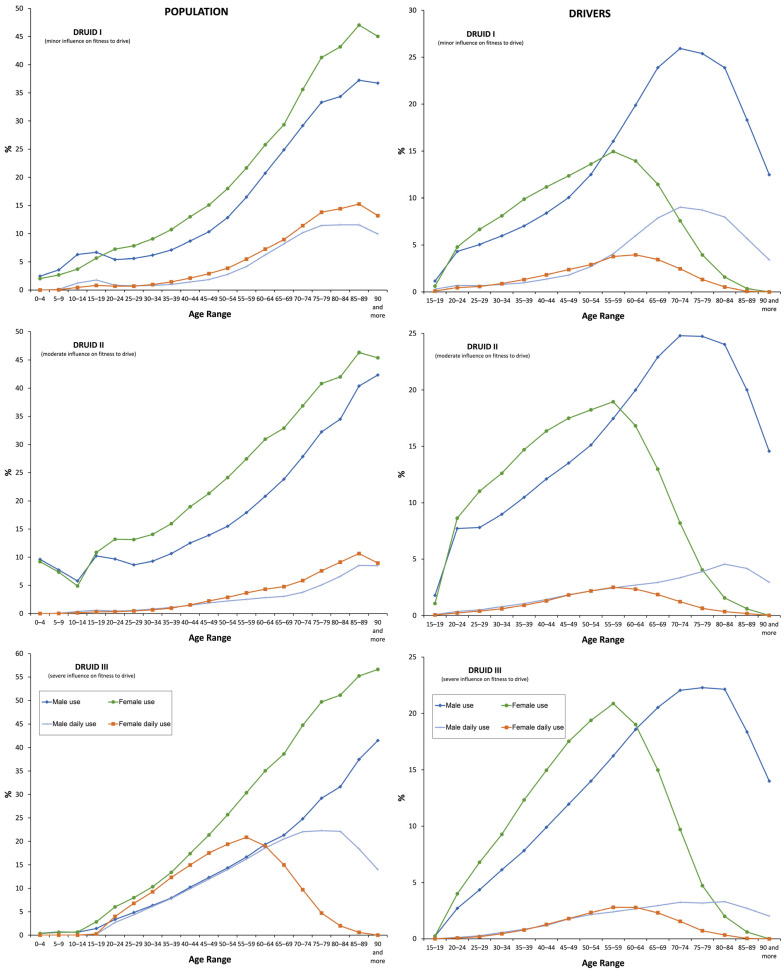
Frequency of the use of DIMs use by DRUID classification.

The yearly use of DIMs increased by approximately 12% (38.60% in 2019 vs. 34.41% in 2015, *p* = 0.001), with a particular impact on acute use, which increased by 35.89% (7.22% in 2019 vs. 5.31% in 2015, *p* = 0.001), followed by chronic use (8.68%, 24.41% in 2019 vs. 22.46% in 2015, *p* = 0.001) and subacute use (4.98%, 6.97% in 2019 vs. 6.64% in 2015, *p* = 0.001) (Figure 3).

The general population preferentially used DRUID II medicines (20.3%), followed by DRUID III medicines (19.08%) and DRUID I medicines (17.11%) (Table 1, Figure 2). Nevertheless, the highest increase in use was observed for DRUID III medicines (19.59%, 20.33% in 2019 vs. 17% in 2015, *p* = 0.001), followed by DRUID I medicines (17.42%, 18.26% in 2019 vs. 15.55% in 2015, *p* = 0.001) and DRUID II medicines (12.93%, 21.91% in 2019 vs. 19.4% in 2015, *p* = 0.001) (Table 2).

Regardless of medicine use, yearly users took 2.27 ± 1.76 DIMs, increasing to 2.87 ± 1.94 for chronic users. Consumption was higher in females than in males, except for the medicines in the DRUID I category (Table 3).

As for daily use, 8.04% of the general population (Table 1) took a mean of 1.35 ± 0.68 DIMs (1.36 ± 0.7 in males vs. 1.35 ± 0.67 in females, *p* = 0.001) (Table 3). The greatest increase in use was observed among daily users (63.95%, 9.10% in 2019 vs. 5.55% in 2015, *p* = 0.001) (Figure 2), which was higher for DRUID II medicines (33.49%, 2.97% in 2019 vs. 2.23% in 2015, *p* = 0.001) than for DRUID III medicines (31.9%, 2.84% in 2019 vs. 2.15% in 2015, *p* = 0.001) and DRUID I medicines (29.3%, 5% in 2015 vs. 3.87 in 2015, *p* = 0.001).

In terms of concomitant use, 46.56% of the general population using a DRUID III medicine also used a DRUID II and 44.74% used a DRUID I medicine. However, with daily use, the concomitant use of DRUID III medicines increased to 61.67% for DRUID II and 52.81% for DRUID I medicines.

Concerning drivers, 27.91% took at least one DIM, which was higher in males than in females (29.02% vs. 26.25%). As in the general population, the use of DIMs was predominantly chronic (16.15%) with a higher use of DRUID II category medicines (15.20%) (Table 1). The use of DIMs increased with age, peaking at 55–59 years for females and 70–74 years for males (Figure 1 and Figure 2, respectively). The increase in the use of DIMs was higher than in the general population, especially in yearly use (17.96%, 29.91% in 2019 vs. 25.35% in 2015, *p* = 0.001) (Figure 3) and in DRUID III medicines use (26.96%, 14.71% in 2019 vs. 11.61% in 2015, *p* = 0.001) (Table 2). Finally, the concomitant use of medicines from different DRUID categories was similar to the general population.

## 3. Discussion

According to our findings, nearly 36% of the general population and 26% of drivers took a DIM from 2015 to 2019. The use of DIMs increased with age and was prevalent among females in the general population and among male drivers. The chronic use and consumption of DRUID II medicines were the most prominent. The general population chronically used about three different DIMs, mainly central nervous system (ATC group N) medicines, especially anxiolytics and opioids, and alimentary tract and metabolism (ATC group A) medicines, especially oral antidiabetics and insulins. In each year of the study period, we detected a relevant concomitant use of different DIMs, such that half of the DRUID III medicines users were also taking a DRUID II and a DRUID I medicine. In addition, the trend of DIMs use was increasing, especially for DRUID III medicines.

To our knowledge, this is the first study to evaluate the use of DIMs according to the DRUID classification. DIMs in the DRUID II category (severe influence on fitness to drive) were the most frequently used, followed closely by DIMs in the DRUID III category (severe influence on fitness to drive), whose use trend showed the greatest increase between 2015 and 2019. These data constitute a serious road safety issue, since many patients, especially drivers, lack sufficient information on how to act when driving vehicles [28].

The use of DIMs, regardless of the DRUID category, is generally higher in females among the general population and in male drivers. This is most likely because in Spain males are the majority behind the wheel. Moreover, the number of driving licenses for females decreases with age [42,43,44,45,46,48].

Like other medicines, DIM use increases with age [49]. These values were predictable, since our region is characterized by an elderly population with an increasing rate of polymedication [50], especially in patients with mental disorders [51], for whom DIMs are mostly indicated.

However, the ability of the elderly to drive safely is a controversial issue. Mental and physical changes accompany aging [52], such as the decline of cognitive, visual, motor, and sensory functions [53]. These factors increase the risk of traffic crashes [52], especially after the age of 80 [54].

In this sense, the Spanish General Regulations for Drivers [55] state that drivers over the age of 65 must renew their license every 5 years, instead of the 10 years required for drivers under the age of 65. In addition, a medical examination to assess the driver’s psychophysical condition is mandatory for license renewal. Related to this, Appendix 4 of these regulations [55] contains a list of diseases that restrict the renewal of a driver’s license. In short, in Spain, the renewal of a driver’s license depends on the state of the driver’s abilities and aptitudes.

The chronic use of DIMs was the most common, with the significant prevalence of daily use. In this sense, the impairment of fitness to drive decreases with chronic and stable medication use [56] due to the tolerance factor [24]. On the other hand, the initiation of treatment, dosage changes, acute use, and multiple daily doses are the most dangerous circumstances for drivers, especially after the age of 50 [8,18,56,57]. The number of medicines consumed should also be considered, as polypharmacy is associated with a higher likelihood of being involved in a traffic crash [57,58]. In the present study, the concomitant use of different DIMs was considerable. This scenario must be considered, especially when using DIMs classified as DRUID II and III (moderate and severe influence on fitness to drive, respectively). This use dramatically increases the risk of a traffic crash, especially if the driver has also consumed alcohol [58].

The present findings seem to be consistent with other European research which has found that central nervous system medicines (ATC group N) are the most commonly consumed [42,58]. Basically, the use of benzodiazepines (anxiolytics), antidepressants and opioid analgesics is predominant and has been increasing in recent years [43,45,46]. The relationship between traffic crashes and the use of benzodiazepines [18,19,20] and opioid analgesics [12,24] is well established, particularly in acute use and with increasing doses. Furthermore, among people taking antidepressants, the effect on fitness to drive is not only due to the influence of the medication but also to the depression symptoms [59,60,61]. However, contradictory data on antidepressants are available in the literature. Medicines such as mirtazapine or tricyclic antidepressants can influence driving, mainly at the beginning of treatment [22,23], although selective serotonin reuptake inhibitors (SSRIs) affect driving only at high doses or when used in combination with other DIMs [62,63,64]. Most of these medicines, except for some types of antidepressants, are classified as DRUID II and DRUID III (severe influence on fitness to drive) [27].

Far behind the above, the next most used DIMs were medicines for the alimentary tract and metabolism (ATC group A). This group includes oral antidiabetics and insulins, which are classified as DRUID I (minor influence on fitness to drive) [27]. These figures, although higher than in other European countries [65], are consistent with a previous study conducted by our group [47] and other national data [66]. Diabetes mellitus is known to affect the fitness to drive due to chronic complications such as retinopathy and neuropathy [67,68] and especially acute complications such as hypoglycemia and hyperglycemia [68,69]. Indeed, hypoglycemia can be a direct consequence of the use of medicines such as insulin [70,71,72] and oral antidiabetic agents such as sulfonylureas and methyglinides [71,73]. Therefore, restrictions have been imposed in Europe and the United States for diabetics to obtain a driver’s license [69,74,75,76].

The DRUID classification was developed by the EU in order to harmonize the criteria for classifying the medicines marketed in Europe according to their influence on fitness to drive (https://www.emcdda.europa.eu/publications/thematic-papers/druid_en, accessed on 25 February 2020). However, this categorization is not without limitations. The DRUID category is assigned based on the assumption that the medicine is used for its primary indication, is administered to an adult, at a normal dose, and at the beginning of the treatment [27]. Nevertheless, the DRUID classification has proven to be suitable for being incorporated into computerized pharmaceutical dispensing systems [77].

Information effectively provided by healthcare professionals, especially physicians and pharmacists, has been shown to have the potential to reduce the annual rate of traffic crashes by up to 45% [28]. In Spain, other warning systems, such as the “medicines and driving” pictogram, have not been fully effective [28]. Therefore, the incorporation of DRUID categorization into prescribing and dispensing tools must be a primary objective for healthcare authorities.

The most important recommendation for physicians and pharmacists is the need to effectively counsel their patients about the effects of DIMs on fitness to drive [78]. According to the conclusions of the DRUID project, health counseling should be brief, individualized, and provided at the time that a DIM is prescribed or dispensed [77]. In addition, following the recommendations of the DRUID project, prescribing/dispensing guidelines should be established based on the use of safer driving medications when available [77].

Specifically, ensuring the safe and effective use of medicines is one of the most important roles of a pharmacist [79]. In this sense, the pharmacist plays an important role in achieving patients’ health goals, monitoring pharmacologic treatment, collaborating with the primary care team, and working in health promotion and disease prevention. Definitely, the pharmaceutical profession is well positioned in the healthcare system and should take the lead in educational programs for the driving population using DIMs.

Finally, the limitations of a retrospective observational design and those inherent to data extraction from health administrative databases should be mentioned. CONCYLIA does not include information on hospital dispensing, consumption derived from private practice, or “over the counter” dispensing. However, this bias is not considered relevant, since nearly 95% of the population in our region is covered by public health insurance and a medical prescription is mandatory for the dispensation of DIMs. Another bias to consider is that CONCYLIA does not include information about whether the patient is a driver. Therefore, a weighting method was established using the driver’s license census, adjusting for age and sex. This method has been extensively tested in previous research by our team [42,43,44,45,46,47,48]. On the other hand, it has been assumed that the individual with a driver’s license is an active driver, but this is not always the case, especially among the elderly. Furthermore, comorbidities in the elderly, such as depression, Parkinson’s disease, insomnia, hypertension, congestive heart failure, osteoarthritis, diabetes mellitus, and others, which certainly affect one’s fitness to drive, were not considered [4]. Lastly, an important limitation is the lack of data on cannabis and marijuana use, which can seriously affect cognition, psychomotor function, and driving performance [80].

## 4. Materials and Methods

This work followed the same methodology as previously described in previous studies conducted by our group [42,43,44,45,46,47,48] and is presented below.

### 4.1. Real-World Study Details

The findings in this manuscript are presented from a population-based registry study following the STROBE [81] (Strengthening the Reporting of Observational Studies in Epidemiology) and RECORD [82] (Reporting of studies Conducted using Observational Routinely-collected Data) recommendations for observational studies. 

**Figure 4:**
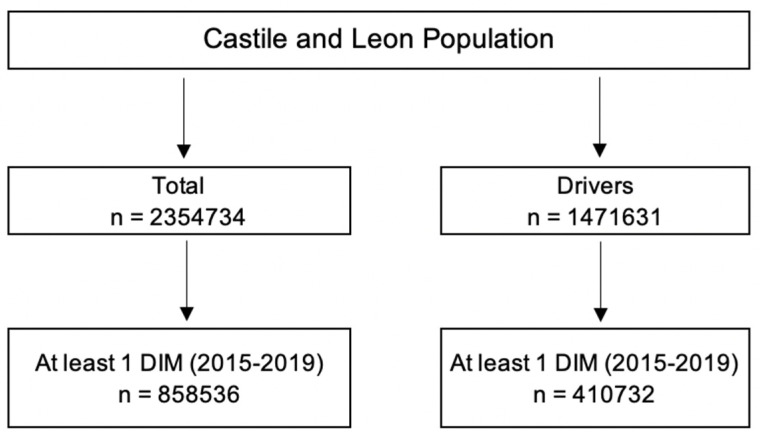
Flowchart of the inclusion of the study population.

All medicines marketed in Spain with the pictogram “medicines and driving” are considered DIMs. In this sense, all DIM dispensations in pharmacies in Castile and Leon from 2015 to 2019 were considered. As in previous manuscripts [42,43,44,45,46,47,48], dispensing was considered to be an approximation of the actual consumption. The total population of Castile and Leon was considered (Table S3). Figure 4 shows the flow chart of the inclusion of the study population.

Data on the use of DIMs were extracted from the Pharmacy Information System of the Castile and Leon Health System, CONCYLIA (http://www.saludcastillayleon.es/ portalmedicamento/es/indicadores informes/concylia, accessed on 3 February 2020). This database includes, but is not limited to: dispensing code and date; pharmacy identification; patient sex and age; generic medicine name; number of packages dispensed; length of treatment; etc. In the CONCYLIA database, medication classification is based on the Anatomical Therapeutic Chemical Code (ATC) [83]. However, CONCYLIA does not collect data on dispensations from hospitals, private practices, and “over the counter medicines”. 

In addition, the National Department of Traffic (Ministry of Interior) (http://www.dgt.es/es/seguridad-vial/estadisticas-e-indicadores/permisos-conduccion/, accessed on 25 January 2020) provided access to drivers’ license census data. Subsequently, as in previous manuscripts [42,43,44,45,46,47,48], the use of DIMs in drivers was adjusted (Table S3).

The Valladolid East Health Area Ethics Committee approved this study on 17 March 2016 (reference number PI 16-387).

### 4.2. Variables

The variables considered were: (1) yearly frequency of the use of DIMs; (2) acute, sub-acute, and chronic use of DIMs; (3) daily use of DIMs; (4) yearly frequency of DIMs use by DRUID categories; and (5) concomitant use of DIMs of diverse DRUID categories.

The length of treatment was determined to be between 1 and 7 days for acute use, between 8 and 29 days for subacute use, and more than 29 days for chronic use. Yearly use is equivalent to the use of at least one DIM during the year.

### 4.3. Statistical Analysis

Sex and age were considered for all analyses. Frequencies (percentages) with their 95% confidence interval (95% CI) or means with their standard deviation (SD) were used to report the results. Differences between the continuous variables were analyzed by the Student’s *t*-test (t), while differences between the categorical variables were analyzed by the Chi-squared test (χ²). The trend in DIM use during the study period was analyzed using the Cochran–Armitage trend test. The statistical significance level was set at *p* ≤ 0.05. Finally, all statistical analyses were performed using the Statistical Package for the Social Sciences (SPSS version 24.0., SPSS Inc, Chicago, IL, USA).

## 5. Conclusions

During the study period, the use of DIMs was frequent and increasing. The use and chronic use of DRUID II and III medicines, which have the greatest impact on fitness to drive, are highlighted. The aging of our region, the high use of DIMs, and polypharmacy increase the risk of having a traffic crash [8,18,56,57,58]. Thus, the knowledge of how medications affect one’s fitness to drive is clearly a relevant road safety issue for patients, physicians, pharmaceutical companies, policymakers, and the general population [84].

The DRUID classification has the necessary characteristics to be useful for physicians and pharmacists to inform drivers about the side effects (cognitive and psychomotor impairment) associated with the use of DIMs [85]. This action is more meaningful for the pharmacist, as they are usually the last opportunity for the patient to be informed before starting treatment [86].

## Figures and Tables

**Table 1:** Use of DIMs according to the CONCYLIA database and the Castile and León drivers’ license census data.

	Population Using DIM % (95CI)					Drivers Using DIM % (95CI)				
	Yearly Use	Daily Use	Type of use			Yearly Use	Daily Use	Type of use		
			Acute	Subacute	Chronic			Acute	Subacute	Chronic
TOTAL DIM										
Total	36.46 (36.4–36.53)	8.04 (8–8.07)	6.44 (6.41–6.47)	6.72 (6.69–6.75)	23.3 (23.25–23.35)	27.91 (27.83–27.98)	5.34 (5.3–5.37)	5.49 (5.46–5.53)	6.26 (6.22–6.3)	16.15 (16.09–16.21)
Male	30.44 (30.35–30.52)	6.52 (6.47–6.56)	6.27 (6.22–6.31)	5.73 (5.69–5.78)	18.44 (18.37–18.51)	29.02 (28.93–29.12)	6.18 (6.13–6.24)	5.65 (5.6–5.69)	5.96 (5.91–6.01)	17.42 (17.34–17.5)
Female	42.28 (42.19–42.37)	9.5 (9.45–9.56)	6.62 (6.57–6.66)	7.67 (7.63–7.72)	27.99 (27.91–28.07)	26.25 (26.13–26.36)	4.08 (4.03–4.13)	5.26 (5.21–5.32)	6.71 (6.65–6.77)	14.27 (14.18–14.36)
	Χ² = 37,645.22; *p* = 0.001	Χ² = 19,471.66; *p* = 0.001	Χ² = 2716.22; *p* = 0.001	Χ² = 3222.11; *p* = 0.001	Χ² = 35,025.11; *p* = 0.001	Χ² = 282,817.29; *p* = 0.001	Χ² = 57,813.63; *p* = 0.001	Χ² = 42,935.41; *p* = 0.001	Χ² = 54,323.97; *p* = 0.001	Χ² = 185,912.23; *p* = 0.001
DRUID I										
Total	17.11 (17.06–17.15)	4.54 (4.51–4.56)	3.68 (3.66–3.71)	2.91 (2.89–2.94)	10.5 (10.46–10.54)	12.43 (12.38–12.48)	3 (2.98–3.03)	3.14 (3.11–3.17)	2.25 (2.22–2.27)	7.04 (7–7.08)
Male	14.3 (14.24–14.36)	3.83 (3.8–3.87)	3.39 (3.35–3.42)	2.3 (2.27–2.32)	8.61 (8.56–8.66)	13.57 (13.5–13.64)	3.6 (3.56–3.64)	3.33 (3.29–3.36)	2.3 (2.27–2.33)	7.94 (7.89–8)
Female	19.82 (19.75–19.89)	5.22 (5.18–5.26)	3.97 (3.93–4)	3.51 (3.48–3.55)	12.33 (12.27–12.39)	10.73 (10.65–10.81)	2.12 (2.08–2.16)	2.86 (2.82–2.9)	2.17 (2.14–2.21)	5.7 (5.64–5.76)
	Χ² = 30,920.70; *p* = 0.001	Χ² = 13,031.53; *p* = 0.001	Χ² = 3233.17; *p* = 0.001	Χ² = 2505.51; *p* = 0.001	Χ² = 29,618.92; *p* = 0.001	Χ² = 142,137.07; *p* = 0.001	Χ² = 38,239.22; *p* = 0.001	Χ² = 21,685.44; *p* = 0.001	Χ² = 25,466.54; *p* = 0.001	Χ² = 96,994.06; *p* = 0.001
DRUID II										
Total	20.3 (20.25–20.35)	2.72 (2.7–2.74)	7.16 (7.13–7.19)	4.12 (4.09–4.14)	9.02 (8.98–9.06)	15.2 (15.15–15.26)	1.79 (1.77–1.81)	5.7 (5.67–5.74)	3.37 (3.34–3.4)	6.13 (6.09–6.17)
Male	16.55 (16.48–16.62)	2.17 (2.14–2.2)	6.21 (6.16–6.25)	3.3 (3.27–3.34)	7.03 (6.99–7.08)	15.43 (15.35–15.5)	2.04 (2.01–2.07)	5.63 (5.58–5.67)	3.24 (3.21–3.28)	6.56 (6.51–6.61)
Female	23.92 (23.84–24)	3.24 (3.21–3.27)	8.08 (8.03–8.13)	4.9 (4.86–4.94)	10.94 (10.88–10.99)	14.87 (14.78–14.96)	1.42 (1.39–1.45)	5.82 (5.76–5.88)	3.57 (3.52–3.62)	5.49 (5.43–5.55)
	Χ² = 18,324.73; *p* = 0.001	Χ² = 6775.92; *p* = 0.001	Χ² = 4413.65.54; *p* = 0.001	Χ² = 2732.69; *p* = 0.001	Χ² = 13,583.15; *P* = 0.001	Χ² = 153,603.72; *p* = 0.001	Χ² = 15,132.36; *p* = 0.001	Χ² = 51,506.85; *p* = 0.001	Χ² = 36,306.531; *p* = 0.001	Χ² = 64,190.65; *p* = 0.001
DRUID III										
Total	19.08 (19.03–19.13)	2.62 (2.59–2.64)	0.99 (0.98–1)	4.58 (4.55–4.6)	13.51 (13.47–13.56)	13.62 (13.56–13.67)	1.69 (1.67–1.71)	0.81 (0.79–0.82)	4.14 (4.11–4.18)	8.67 (8.62–8.71)
Male	13.27 (13.21–13.33)	1.83 (1.81–1.86)	0.76 (0.74–0.77)	3.46 (3.43–3.5)	9.05 (9–9.1)	13.27 (13.2–13.34)	1.84 (1.81–1.87)	0.75 (0.73–0.77)	3.73 (3.69–3.77)	8.79 (8.73–8.85)
Female	24.69 (24.61–24.76)	3.37 (3.34–3.4)	1.22 (1.2–1.23)	5.65 (5.61–5.69)	17.82 (17.75–17.89)	14.13 (14.04–14.22)	1.48 (1.45–1.51)	0.9 (0.87–0.92)	4.76 (4.7–4.81)	8.48 (8.41–8.55)
	Χ² = 13,929.87; *p* = 0.001	Χ² = 7836.51; *p* = 0.001	Χ² = 708.04; *p* = 0.001	Χ² = 648.79; *p* = 0.001	Χ² = 12,536.37; *p* = 0.001	Χ² = 139,899.19; *p* = 0.001	Χ² = 12,872.79; *p* = 0.001	Χ² = 8304.99; *p* = 0.001	Χ² = 34,128.12; *p* = 0.001	Χ² = 94,925.86; *p* = 0.001

Abbreviations: DIM, driving-impairing medicines, 95CI, confidence interval.

**Table 2:** Evolution of the use of DIMs according to CONCYLIA database and the Castile and León drivers’ license census data (2015–2019).

	Population Using DIM % (95CI)					Drivers Using DIM % (95CI)				
	2015	2016	2017	2018	2019	2015	2016	2017	2018	2019
DRUID category										
I										
Total	15.55 (15.5–15.59)	17.14 (17.09–17.19)	16.73 (16.69–16.78)	17.85 (17.81–17.9)	18.26 (18.21–18.31)	11.02 (10.97–11.07)	12.41 (12.36–12.47)	12.24 (12.18–12.29)	13.03 (12.97–13.08)	13.45 (13.39–13.5)
Male	12.69 (12.63–12.76)	14.23 (14.17–14.29)	13.97 (13.9–14.03)	15.09 (15.02–15.15)	15.52 (15.45–15.58)	12.01 (11.95–12.08)	13.5 (13.43–13.58)	13.4 (13.33–13.47)	14.34 (14.27–14.42)	14.6 (14.53–14.67)
Female	18.31 (18.24–18.38)	19.95 (19.88–20.02)	19.41 (19.34–19.48)	20.53 (20.46–20.6)	20.89 (20.82–20.97)	9.51 (9.44–9.59)	10.77 (10.69–10.85)	10.51 (10.43–10.59)	11.1 (11.02–11.18)	11.76 (11.67–11.84)
	Χ² = 6242.63; *p* = 0.001	Χ² = 6220.82; *p* = 0.001	Χ² = 6371.31; *p* = 0.001	Χ² = 6240.85; *p* = 0.001	Χ² = 6243.2; *p* = 0.001	Χ² = 26,691.39; *p* = 0.001	Χ² = 28,349.01; *p* = 0.001	Χ² = 27,655.09; *p* = 0.001	Χ² = 29,166.77; *p* = 0.001	Χ² = 30,466.79; *p* = 0.001
II										
Total	19.4 (19.35–19.45)	21.07 (21.02–21.12)	18.68 (18.63–18.73)	20.43 (20.38–20.49)	21.91 (21.86–21.97)	14.02 (13.96–14.07)	15.52 (15.46–15.58)	14.23 (14.17–14.28)	15.72 (15.66–15.78)	16.54 (16.48–16.6)
Male	15.84 (15.77–15.9)	17.34 (17.27–17.41)	14.98 (14.91–15.04)	16.66 (16.59–16.73)	17.94 (17.87–18.01)	14.53 (14.46–14.6)	15.82 (15.75–15.9)	14.26 (14.18–14.33)	15.89 (15.82–15.97)	16.64 (16.56–16.72)
Female	22.84 (22.77–22.92)	24.68 (24.6–24.75)	22.27 (22.19–22.34)	24.08 (24.01–24.16)	25.74 (25.66–25.81)	13.24 (13.15–13.32)	15.06 (14.97–15.15)	14.18 (14.09–14.27)	15.47 (15.37–15.56)	16.38 (16.29–16.48)
	Χ² = 4212.31; *p* = 0.001	Χ² = 3734.73; *p* = 0.001	Χ² = 3259.54; *p* = 0.001	Χ² = 3234.94; *p* = 0.001	Χ² = 4093.43; *p* = 0.001	Χ² = 29,478.34; *p* = 0.001	Χ² = 32,470.1; *p* = 0.001	Χ² = 28,803.92; *p* = 0.001	Χ² = 30,527.18; *p* = 0.001	Χ² = 32,463.86; *p* = 0.001
III										
Total	17 (16.95–17.04)	19.17 (19.12–19.22)	18.85 (18.8–18.9)	20.05 (20–20.1)	20.33 (20.28–20.38)	11.61 (11.56–11.66)	13.65 (13.6–13.71)	13.57 (13.52–13.63)	14.51 (14.46–14.57)	14.74 (14.68–14.79)
Male	11.52 (11.46–11.58)	13.27 (13.21–13.34)	13.02 (12.96–13.08)	14.14 (14.08–14.21)	14.39 (14.33–14.46)	11.49 (11.42–11.56)	13.24 (13.17–13.31)	13.15 (13.07–13.22)	14.18 (14.11–14.25)	14.3 (14.23–14.38)
Female	22.29 (22.22–22.37)	24.87 (24.79–24.94)	24.48 (24.4–24.56)	25.76 (25.68–25.84)	26.04 (25.96–26.12)	11.8 (11.72–11.88)	14.28 (14.19–14.36)	14.21 (14.12–14.3)	15 (14.91–15.1)	15.38 (15.28–15.47)
	Χ² = 3115.65; *p* = 0.001	Χ² = 2544.38; *p* = 0.001	Χ² = 2879.54; *p* = 0.001	Χ² = 2704.39; *p* = 0.001	Χ² = 2772.62; *p* = 0.001	Χ² = 24,930.35; *p* = 0.001	Χ² = 28,892.67; *p* = 0.001	Χ² = 27,808.57; *p* = 0.001	Χ² = 29,052.18; *p* = 0.001	Χ² = 29,466.38; *p* = 0.001

Abbreviations: DIM, driving-impairing medicines, 95CI, confidence interval.

**Table 3:** Average DIMs consumed in Castile and Leon by frequency of use.

Frequency of Use	DRUID Category	Population Using DIM (Mean ± SD)				Drivers Using DIM(Mean ± SD)			
		Males	Females	TOTAL	t, *p*	Males	Females	TOTAL	t, *p*
Acute	DRUID I	1.02 ± 0.13	1.02 ± 0.15	1.02 ± 0.14	t = −3.21; *p* = 0.078	1.02 ± 0.13	1.02 ± 0.14	1.02 ± 0.13	t = −5.27; *p* = 0.093
	DRUID II	1.03 ± 0.17	1.04 ± 0.19	1.03 ± 0.18	t = −23.70; *p* = 0.001	1.03 ± 0.17	1.03 ± 0.19	1.03 ± 0.18	t = −79.87; *p* = 0.043
	DRUID III	1 ± 0.06	1 ± 0.05	1 ± 0.05	t = 1.28; *p* = 0.199	1 ± 0.05	1 ± 0.04	1 ± 0.05	t = 3.51; *p* = 0.134
	TOTAL	1.03 ± 0.19	1.04 ± 0.21	1.03 ± 0.2	t = −21.9; *p* = 0.001	1.03 ± 0.19	1.04 ± 0.2	1.03 ± 0.19	t = −11.9; *p* = 0.001
Sub-acute	DRUID I	1.12 ± 0.34	1.12 ± 0.35	1.12 ± 0.35	t = −2.34; *p* = 0.123	1.12 ± 0.35	1.14 ± 0.36	1.13 ± 0.35	t = −7.89; *p* = 0.001
	DRUID II	1.25 ± 0.49	1.32 ± 0.55	1.29 ± 0.53	t = −42.65; *p* = 0.001	1.25 ± 0.49	1.32 ± 0.55	1.28 ± 0.51	t = −31.06; *p* = 0.001
	DRUID III	1.08 ± 0.29	1.11 ± 0.32	1.1 ± 0.31	t = −25.87; *p* = 0.001	1.08 ± 0.28	1.1 ± 0.31	1.09 ± 0.3	t = −17.77; *p* = 0.001
Chronic	TOTAL	1.37 ± 0.61	1.44 ± 0.66	1.41 ± 0.64	t = −44.32; *p* = 0.001	1.38 ± 0.61	1.43 ± 0.65	1.4 ± 0.63	t = −28.8; *p* = 0.001
	DRUID I	1.64 ± 0.92	1.58 ± 0.9	1.6 ± 0.91	t = 31.810; *p* = 0.001	1.65 ± 0.93	1.46 ± 0.79	1.59 ± 0.89	t = 75.72; *p* = 0.001
	DRUID II	1.9 ± 1.26	1.98 ± 1.25	1.95 ± 1.25	t = −29.71; *p* = 0.001	1.95 ± 1.31	2.06 ± 1.37	1.99 ± 1.33	t = −27.55; *p* = 0.001
	DRUID III	1.56 ± 0.87	1.68 ± 0.94	1.64 ± 0.92	t = −74.74; *p* = 0.001	1.58 ± 0.88	1.7 ± 0.97	1.63 ± 0.92	t = −53.21; *p* = 0.001
	TOTAL	2.66 ± 1.84	3 ± 1.99	2.87 ± 1.94	t = −44.32; *p* = 0.001	2.69 ± 1.87	2.92 ± 2.01	2.77 ± 1.92	t = −65.66; *p* = 0.001
Yearly	DRUID I	1.41 ± 0.78	1.39 ± 0.77	1.4 ± 0.77	t = 15.09; *p* = 0.001	1.4 ± 0.78	1.28 ± 0.64	1.36 ± 0.74	t = 84.04; *p* = 0.001
	DRUID II	1.44 ± 0.95	1.52 ± 0.99	1.49 ± 0.97	t = −62.85; *p* = 0.001	1.46 ± 0.98	1.48 ± 0.99	1.47 ± 0.99	t = −9.29; *p* = 0.001
	DRUID III	1.4 ± 0.77	1.51 ± 0.85	1.47 ± 0.83	t = −94.53; *p* = 0.001	1.4 ± 0.77	1.46 ± 0.83	1.43 ± 0.8	t = −31.72; *p* = 0.001
	TOTAL	2.06 ± 1.61	2.404 ± 1.83	2.27 ± 1.76	t = −204.39; *p* = 0.001	2.07 ± 1.64	2.15 ± 1.73	2.10 ± 1.67	t = −32.08; *p* = 0.001

Abbreviations: DIM, driving-impairing medicines, SD, standard deviation.

## Data Availability

Restrictions apply to the availability of these data. Data were obtained from regional health authorities (Gerencia Regional de Salud (GRS)) and may be requested from conciertofco@saludcastillayleon.es (GRS).

## Supplementary Materials

The following supporting information can be downloaded at: https://www.mdpi.com/article/10.3390/ph16040508/s1, Table S1: Definition of DRUID categories; Table S2: List of the 20 most consumed DIMs according to DRUID classification in Castile and Leon during the study period (packages/year); Table S3: Evolution of the Castile and Leon population and driver’s licenses (2015–2019).
