# Supplementary material for: Trends in the Use of Driving-Impairing Medicines According to the DRUID Category: A Population-Based Registry Study with Reference to Driving in a Region of Spain between 2015 and 2019
Source: Pharmaceuticals (Basel). 2023 Mar 29;16(4):508. doi: 10.3390/ph16040508 (PMC10145018; doi:10.3390/ph16040508)
Supplement: Supplementary file 1 [file pharmaceuticals-16-00508-s001.zip › pharmaceuticals-2266604-supplementary.pdf]

Table S1. Definition of DRUID categories

Data to be used for assigning category	DRUID CATEGORIES		
	MINOR INFLUENCE	MODERATE INFLUENCE	MAJOR INFLUENCE
Pharmacodynamic and kinetic data	No influence expected	Moderate influence expected	Severe influence expected
Pharmacovigilance data	Some demonstrate of CNS side effects or unwanted effects that impairing driving	Demonstration of CNS side effects (not severe) or unwanted effects that impairing driving	Demonstration of CNS side effects (severe) or unwanted effects that impairing driving
Experimental and epidemiology data	Some impairment in some experimental studies. Slight increased risk demonstrated in epidemiological studies.	Impairment of driving performance is seen in various experimental studies. In epidemiological studies a significant increased risk is demonstrated.	Gross impairment of driving performance or performance related to driving is repeatedly seen. In epidemiological studies a significant and meaningful increased risk is demonstrated.
Additional data	Some data on possible impairment.	Various data on impairment (not severe).	Data on severe impairment.

Table S2. List of the 20 most consumed DIMs according to DRUID classification in Castile and Leon during the study period (packages/year).

DRUID I			DRUID II			DRUID III		
**Code ATC**	**Name**	**Packages**	**Code ATC**	**Name**	**Packages**	**Code ATC**	**Name**	**Packages**
N02BB02	Metamizole sodium	950616	N02AJ06	Codeine and paracetamol	242350	N05BA06	Lorazepam	892805
N06AB10	Escitalopram	256906	N02BF02	Pregabalin	237229	N05BA12	Alprazolam	780861
N06AB06	Sertraline	211002	N05CF02	Zolpidem	225084	N02AJ13	Tramadol and paracetamol	616769
A10BD07	Metformin and sitagliptin	185756	N06AX16	Venlafaxine	213957	N05CD06	Lormetazepam	512782
N06AB05	Paroxetine	175318	N06AX21	Duloxetine	173940	N05BA08	Bromazepam	358449
A10BD08	Metformin and vildagliptin	158585	N05BA05	Potassium clorazepate	150847	N05BA01	Diazepam	234307
A10AE04	Insuline glargine	155976	N05AH04	Quetiapine	126580	N06AX11	Mirtazapine	210905
N06AB04	Citalopram	88208	N02AB03	Fentanyl	119808	N06AA09	Amitriptyline	133467
A10BB09	Glicazide	83782	R03DA12	Mepyramine theophyllinacetate	117311	N02AX02	Tramadol	122596
N06AB03	Fluoxetine	82129	N03AX14	Levetiracetam	112675	N06AX05	Trazodone	101427
A10BX02	Repaglinide	74773	N05AH03	Olanzapine	89232	N05BA10	Ketazolam	49319
N06BA04	Methylphenidate	68203	N03AE01	Clonazepam	85763	N02AJ14	Tramadol and dexketoprofen	33038
A10BH01	Sitagliptin	66598	N03AG01	Valproic acid	80592	N05AX13	Paliperidone	26188
A10BH05	Linagliptin	60347	N05AL01	Sulpiride	80559	N05AA02	Levomepromazine	25666
A10BB12	Glimepiride	42670	N06AX23	Desvenlafaxine	74868	N06AX03	Mianserine	18223
N05CM02	Clomethiazole	41229	N03AX12	Gabapentine	72669	N05CD11	Loprazolam	17339
P01BA02	Hydroxychloroquine	40461	N04BA02	Levodopa and decarboxylase inhibitor	69619	N05CF01	Zoplicone	16929
A10BK01	Dapagliflozin	34716	N03AX09	Lamotrigine	62862	N05AX08	Risperidone intravenous	15688
A10AB05	Insulin aspart	33020	N06DA02	Donepezil	58355	N03AA02	Phenobarbital	14661
N01BB02	Lidocaine	32392	N05AX08	Risperidone	58005	N02AB03	Fentanyl	13607

Abbreviations: ATC, Anatomic Therapeutic Chemical

Table S3: Evolution of the Castile and Leon population and driver´s licenses (2015-2019).

Rank age	Population														
	2015			2016			2017			2018			2019		
	Male	Female	Total	Male	Female	Total	Male	Female	Total	Male	Female	Total	Male	Female	Total
0-4	45.405	42.504	87.909	44.382	41.386	85.768	42.905	40.144	83.049	41.604	39.121	80.725	40.059	38.097	78.156
5-9	50.925	48.078	99.003	50.665	47.821	98.486	50.035	47.344	97.379	48.594	45.657	94.251	47.764	44.531	92.295
10-14	49.439	47.220	96.659	49.847	47.730	97.577	50.316	48.259	98.575	51.124	48.407	99.531	51.486	48.715	100.201
15-19	48.620	46.904	95.524	48.862	46.935	95.797	48.939	46.706	95.645	49.610	47.885	97.495	50.274	48.195	98.469
20-24	54.724	53.382	108.106	53.230	52.333	105.563	52.182	51.246	103.428	51.428	50.777	102.205	51.319	51.186	102.505
25-29	62.787	61.247	124.034	61.109	59.382	120.491	59.522	57.531	117.053	58.298	56.506	114.804	56.603	54.511	111.114
30-34	75.089	71.664	146.753	71.742	68.841	140.583	68.575	66.051	134.626	65.942	63.241	129.183	62.041	60.593	122.634
35-39	90.372	87.031	177.403	87.267	83.676	170.943	83.600	80.400	164.000	79.663	76.944	156.607	74.317	72.988	147.305
40-44	92.686	89.879	182.565	92.967	90.094	183.061	92.799	89.681	182.480	92.434	89.499	181.933	89.068	87.308	176.376
45-49	93.082	91.643	184.725	93.035	91.392	184.427	92.076	90.588	182.664	91.744	89.952	181.696	90.343	88.978	179.321
50-54	93.252	90.618	183.870	93.251	91.395	184.646	93.500	91.893	185.393	92.913	92.426	185.339	91.810	91.309	183.119
55-59	87.280	84.212	171.492	88.988	85.894	174.882	89.831	86.956	176.787	90.852	87.988	178.840	91.071	89.305	180.376
60-64	72.448	69.337	141.785	75.073	72.029	147.102	77.520	74.875	152.395	79.640	77.583	157.223	82.509	80.561	163.070
65-69	65.430	66.777	132.207	66.403	67.268	133.671	67.615	68.053	135.668	67.660	67.825	135.485	68.270	67.745	136.015
70-74	56.526	61.968	118.494	58.076	63.396	121.472	58.913	64.067	122.980	60.320	64.908	125.228	60.713	65.590	126.303
75-79	45.154	56.939	102.093	43.540	53.807	97.347	43.510	52.990	96.500	46.205	55.251	101.456	49.158	58.079	107.237
80-84	44.543	62.354	106.897	44.319	62.772	107.091	42.312	60.175	102.487	39.465	56.065	95.530	36.642	51.825	88.467
85-89	27.547	46.335	73.882	28.618	47.555	76.173	29.407	48.337	77.744	29.731	48.690	78.421	30.015	48.916	78.931
≥ 90	13.282	30.034	43.316	14.119	31.809	45.928	14.662	32.970	47.632	15.333	34.407	49.740	15.991	35.885	51.876
**Total**	**1.168.591**	**1.208.126**	**2.376.717**	**1.165.493**	**1.205.515**	**2.371.008**	**1.158.219**	**1.198.266**	**2.356.485**	**1.152.560**	**1.193.132**	**2.345.692**	**1.139.453**	**1.184.317**	**2.323.770**

Rank age	Driver´s licence census														
	2015			2016			2017			2018			2019		
	Male	Female	Total	Male	Female	Total	Male	Female	Total	Male	Female	Total	Male	Female	Total
15-19	9.282	5.586	14.868	9.238	5.634	14.872	8.357	4.689	13.046	8.702	5.102	13.804	8.702	5.102	13.804
20-24	43.294	35.387	78.681	42.165	34.280	76.445	40.859	33.207	74.066	39.837	32.570	72.407	39.837	32.570	72.407
25-29	55.831	50.618	106.449	53.617	48.755	102.372	51.913	46.861	98.774	50.269	45.329	95.598	50.269	45.329	95.598
30-34	69.810	61.387	131.197	66.192	58.489	124.681	62.677	56.134	118.811	59.765	53.560	113.325	59.765	53.560	113.325
35-39	86.841	75.838	162.679	83.112	72.880	155.992	79.204	70.129	149.333	74.693	66.771	141.464	74.693	66.771	141.464
40-44	89.294	76.277	165.571	88.673	76.856	165.529	88.025	76.717	164.742	87.163	76.513	163.676	87.163	76.513	163.676
45-49	90.151	74.310	164.461	89.641	74.389	164.030	88.611	74.242	162.853	87.961	74.534	162.495	87.961	74.534	162.495
50-54	90.450	67.282	157.732	90.290	69.036	159.326	90.158	70.695	160.853	89.420	72.062	161.482	89.420	72.062	161.482
55-59	85.820	56.346	142.166	87.607	59.781	147.388	88.543	62.443	150.986	89.826	64.796	154.622	89.826	64.796	154.622
60-64	71.450	36.255	107.705	74.219	40.173	114.392	76.748	44.338	121.086	79.194	48.281	127.475	79.194	48.281	127.475
65-69	62.572	23.964	86.536	63.824	26.068	89.892	65.577	28.466	94.043	65.978	30.748	96.726	65.978	30.748	96.726
70-74	51.161	12.390	63.551	52.595	13.755	66.350	53.663	15.096	68.759	55.094	16.442	71.536	55.094	16.442	71.536
75-79	35.993	5.000	40.993	34.852	5.277	40.129	35.960	6.008	41.968	38.829	7.105	45.934	38.829	7.105	45.934
80-84	28.304	1.941	30.245	28.194	2.055	30.249	27.809	2.319	30.128	26.500	2.491	28.991	26.500	2.491	28.991

85-89	14.160	429	14.589	14.133	484	14.617	14.548	588	15.136	14.814	661	15.475	14.814	661	15.475
≥ 90	2.944	22	2.966	5.669	50	5.719	6.176	77	6.253	7.350	112	7.462	7.350	112	7.462
**Total**	**887.357**	**583.032**	**1.470.389**	**884.021**	**587.962**	**1.471.983**	**878.828**	**592.009**	**1.470.837**	**875.395**	**597.077**	**1.472.472**	**875.395**	**597.077**	**1.472.472**
